# Difficult Dentition

**Published:** 1860-02

**Authors:** N. S. Bancroft


### DIFFICULT DENTITION.

BY N. S. BANCROFT.

Read before the Michigan Dental Association, of Detroit, Jan. 10th, 1860.

On the subject of Difficult Dentition: I suppose, if parents were absolutely healthy, there would be but little trouble with the children in dentition. Let the child be properly begotten and fed—let the habits of both mother and child be correct, and dentition will go on without serious trouble. Hence the treatment should begin with the mother, long before the birth of the child, and be continued after, and then, if the child does suffer, whatever else we do, let us not poison it.

The principal danger to be apprehended, is from a confined state of the bowels: so I would have the child fed, (or the mother, in case the child derives its food exclusively from her,) in such a manner as to keep the bowels free, and there is no danger.

If the child manifests febrile symptoms, I would bathe it. "Cold water, doctor," do you say? No, I would use no cold water, except ice to the gums, if they be much inflamed. Inclose a small bit of ice in "a rag" and put in the mouth a "sugar teat" without the sugar.

I am aware that the doctrine above announced in regard to the state of the bowels is not according to the books or general notions; yet I apprehend the principal danger in a relaxed state of the alvine functions, consists in the treatment usually instituted for it. With my experience and observation in these cases, I rely entirely on diet and bathing, as the only safe treatment.

On arresting decay in teeth. Allow me to call your attention to a new compound, (amalgam) which Dr. Douglass is using for plugs. I have had opportunities of examining some of these plugs after they have been inserted some time—they do not appear to tarnish easily, and are very hard, and of course make a good masticating surface. It seems a pity that a plugging material, whose merits in some respects are superior to gold, should be limited to secret practice, when humanity is suffering for the want of just such a boon—and particularly Dentists, are obliged to work away with gold, when it would be so easy, if they only knew this other. Pity it is not patented, so we could buy it.

